# Supplementary material for: Association between oral microbiome and seven types of cancers in East Asian population: a two-sample Mendelian randomization analysis
Source: Front Mol Biosci. 2023 Nov 21;10:1327893. doi: 10.3389/fmolb.2023.1327893 (PMC10702768; doi:10.3389/fmolb.2023.1327893)
Supplement: Supplementary file 1 [file DataSheet1.ZIP › Supplementary Materials/MR plots of saliva/saliva═╝/Prostate cancer/pheno.2672_to_prostate cancer_forest.pdf]

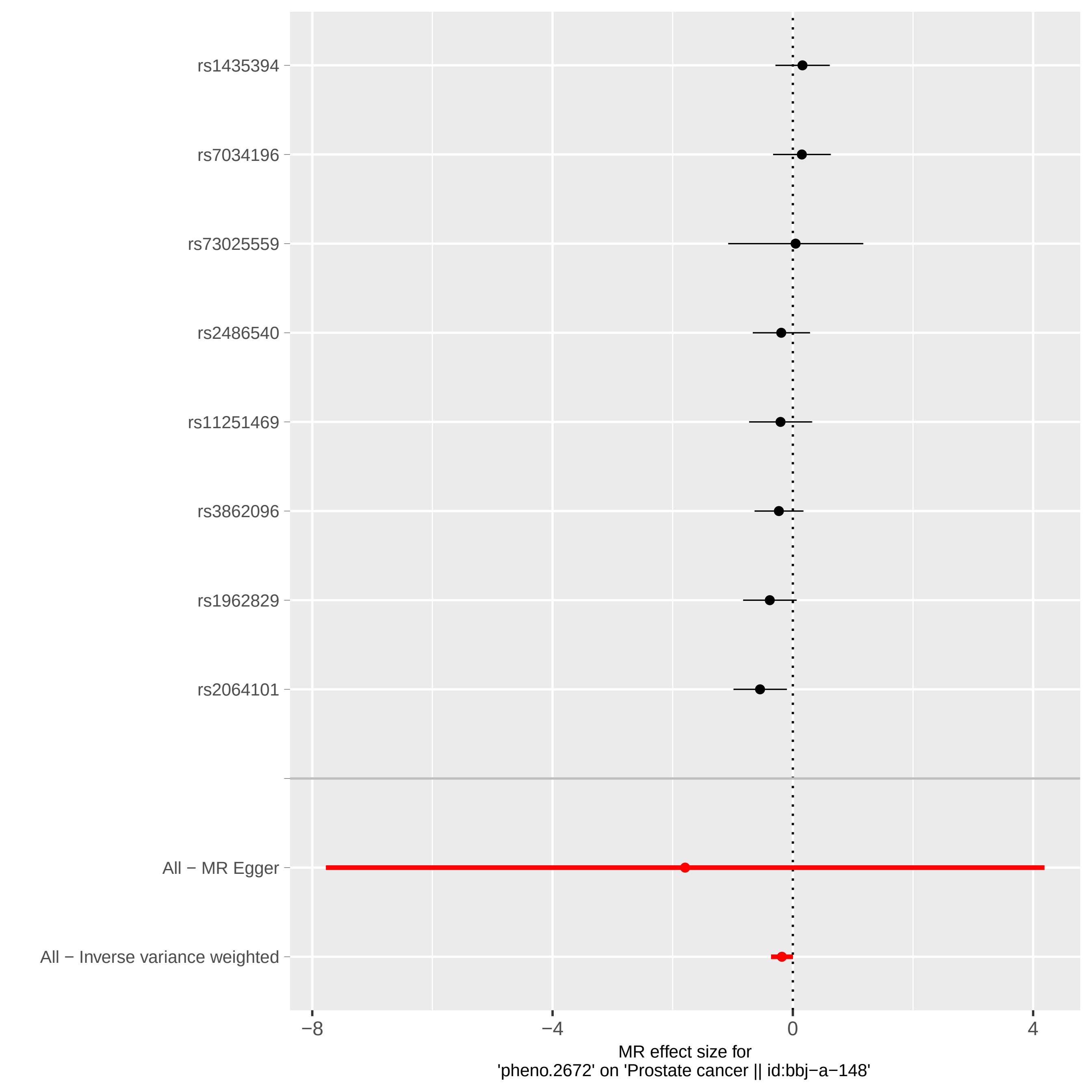
rs1435394
rs7034196
rs73025559
rs2486540
rs11251469
rs3862096
rs1962829
rs2064101
All − MR Egger
All − Inverse variance weighted
−8
−4
0
4
MR effect size for
'pheno.2672' on 'Prostate cancer || id:bbj−a−148'
